# Anti-Glioma Agents and Hyperbaric Oxygen for Prospective Therapeutic Approaches

**DOI:** 10.3390/ijms27198504

**Published:** 2026-09-23

**Authors:** Emanuela B. Pucko, Jakub Godlewski, Natalia Kaźmierczak, Robert P. Ostrowski

**Affiliations:** 1 Department of Neurooncology, Mossakowski Medical Research Institute, Polish Academy of Sciences (MMRI-PAS), 02-106 Warsaw, Poland; epucko@imdik.pan.pl (E.B.P.); jgodlewski@imdik.pan.pl (J.G.); 2 Faculty of Biotechnology and Food Sciences, Łódź University of Technology, 90-530 Lodz, Poland; 246993@edu.p.lodz.pl

**Keywords:** glioblastoma, glioma stem cells, hyperbaric oxygen, combination therapy, phytopharmaceuticals, neuroinflammation

## Abstract

Adjuvant treatments for IDH-wild-type adult high-grade gliomas (HGGs) are being explored to improve therapeutic efficacy. Tumor hypoxia poses a major therapeutic challenge. In this narrative review, we discuss the potential of hyperbaric oxygen to counteract hypoxia and therapeutic resistance in HGG, with particular emphasis on glioblastoma. We delineate the pros and cons of combining treatment with hyperbaric oxygen (HBO) with established and experimental treatments for glioblastoma. HBO has been investigated as an adjuvant treatment alongside radiotherapy and chemotherapy, including new investigational compounds and approaches, with predominantly favorable outcomes in overall treatment efficacy. Among the numerous anticancer phytopharmaceuticals and nutraceuticals in this treatment paradigm, research on curcumin and its derivatives may lay the groundwork for promising future research directions and new chemical syntheses. The growing body of laboratory and clinical data suggests that HBO should be considered a promising component of modern oncological strategies for patients with HGG. However, further studies are needed to substantiate the effects of HBO since it has also been associated with increased tumor proliferation in some studies. Moreover, further studies are needed to substantiate the effects of HBO on tumor/immune interactions, stemness, therapy resistance, and patients’ quality of life.

## 1. Introduction

Oncological diseases currently represent one of the most formidable challenges in contemporary medicine, serving as a primary driver of global mortality. According to 2019 WHO data, malignant neoplasms are the leading or secondary cause of death in populations under the age of 70 in 112 out of 183 analyzed countries [1]. The etiology of neoplastic processes, including glial tumors, is multifactorial. Key determinants of oncogenesis include, among others, behavioral factors such as lifestyle and health habits; environmental factors, including progressive industrialization and systemic exposure to xenobiotics with carcinogenic potential; and radiation. Both electromagnetic and ionizing radiation play significant roles, the latter being a recognized risk factor with a well-documented mechanism for inducing neoplastic transformations [2].

Within the spectrum of diseases characterized by the highest degree of biological aggression, glioblastoma, IDH-wildtype, occupies a distinct position, classified as a CNS WHO Grade 4 malignancy [3,4]. The annual incidence rate is approximately 3 per 100,000 people in developed countries, and the median age of diagnosis is 64 years [5]. The root of the disease remains unknown. Nevertheless, radiation previously used to treat other cancers and conditions, or for diagnostic applications, has been observed to be a contributing factor [6,7,8,9]. Its histopathological profile—dominated by pleomorphic cells, multinucleated forms, and extensive foci of necrosis—accounts for the disease’s fulminant clinical course. The clinical manifestation of glioblastoma (GBM), which includes persistent headaches, escalating intracranial hypertension, and progressive cognitive decline, is a direct function of the tumor’s anatomical localization. Despite the use of advanced diagnostic imaging modalities, such as Magnetic Resonance Imaging (MRI) and Positron Emission Tomography (PET), and the implementation of innovative therapeutic strategies—ranging from robotic systems to immunotherapy and modern drug carriers—prognoses for GBM remain dismal [10,11]. Several factors contribute to the challenges of treating glioblastoma. Firstly, the diagnosis of the tumor is relatively late, by which time it has grown significantly. Like all drugs that affect brain cells, chemotherapy compounds have difficulty crossing the blood–brain barrier. The primary obstacle to the efficacy of the standard-of-care protocol, which combines surgical resection with radio- and chemotherapy, is the diffuse growth pattern of the neoplasm. Infiltration of the healthy parenchyma often renders a total gross resection technically infeasible. Furthermore, localization within eloquent brain regions further constrains surgical radicality, inevitably leading to disease recurrence [3].

Despite advancements in neurosurgical techniques, comprehensive management of glioblastoma patients is expected to combine operative methods, innovative systemic therapies, and advanced care planning [12]. In recent years, the standard of care has evolved significantly, primarily due to the introduction of Temozolomide (TMZ) in combination with surgery and radiation [13]. In cases of primary treatment failure, supplementary options and salvage therapies become critical. These include the administration of bevacizumab, lomustine, carmustine, and the multi-drug PCV regimen (procarbazine, lomustine, and vincristine) [14]. Moreover, the implementation of Tumor-Treating Fields (TTFields) technology, which utilizes low-intensity, intermediate-frequency alternating electric fields, represents significant progress in adjuvant therapy, extending patients’ survival by several months on average and more than doubling the 5-year survival rate compared to chemotherapy alone [15]. Importantly, this modality exhibits no systemic toxicity and does not exacerbate adverse effects when used in combination with other modalities. Potential side effects are typically limited to mild, reversible integumentary reactions at the electrode application sites [15,16].

Evidence suggests that the mechanism of action of TTFields involves inhibiting cellular division during mitosis and inducing apoptosis in neoplastic cells. This translates into a measurable prolongation of patient survival while maintaining a high safety profile [17]. This therapy is characterized by a unique pharmacological profile: its efficacy is not constrained by the Blood–Brain Barrier (BBB) or the classical pharmacokinetic processes that often limit the efficacy of traditional chemotherapy. Difficulty remains posed by non-supratentorial glioblastoma locations, although some progress has been made in approaches to targeting deeply located malignant gliomas [18,19]. Nevertheless, further research on subtentorial tumors treated with TTFields is warranted [20].

Taking the above into account, it seems that an additional component of antiglioma therapies could be considered to enhance therapeutic efficacy. Therefore, we briefly review the hypoxic aspect of glioblastoma tumor biology that creates a major conduit for therapy failure. Further, we consider the pros and cons of hyperbaric oxygen therapy as a modality for treating HGGs, as corroborated by relevant research reports, including combination therapy with HBO and current established treatments, as well as several experimental treatments for glioblastoma. Finally, we discuss the therapeutic potential of new chemical syntheses and selected phyto- and nutraceuticals in this context and outline promising future directions.

The search strategy for this narrative review was developed to identify relevant studies investigating the therapeutic role of hyperbaric oxygen (HBO) combined with radiotherapy, chemotherapy, and phytopharmaceuticals for glioblastoma. A comprehensive and systematic literature search was executed between 3 October 2025, and 1 July 2026, by all authors. The following electronic bibliographic databases were searched from 1999 onwards: PubMed/MEDLINE, Embase, Scopus, Cochrane Central Register of Controlled Trials, and Web of Science. The search strategy was developed by the authors using a combination of Medical Subject Headings (MeSH/Emtree) terms and free-text keywords, including glioblastoma, curcumin, artemisinin, temozolomide, as well as hyperbaric oxygen, high grade glioma, glioma stem cells, adjuvant therapy, etc., which were added to searches using appropriate operators. The core conceptual framework was structured around five primary domains using Boolean operators: Glioblastoma, Hyperbaric Oxygen, Radiotherapy, Chemotherapy, and Phytopharmaceuticals. To ensure literature saturation, secondary search strategies were implemented: reference lists of all eligible primary studies and relevant previous reviews were manually screened. Included were predominantly reports on patients with malignant gliomas and preclinical model data, all of which concerned combination therapies relevant to the study subject. Reports on non-malignant tumors or written in a language other than English were excluded. Additionally, gray literature and ongoing clinical trials were explored via Google Scholar, Scopus, ClinicalTrials.gov, and the World Health Organization International Clinical Trials Registry Platform. To ensure comprehensiveness, grey literature, including conference abstracts and institutional website data, was also screened. Conference abstracts were included only if they provided explicit quantitative outcomes. Commercial websites were avoided, whereas those from academia, government agencies, or reputable societies that contained scientific references were preferred. Since those sources lack the standard peer review, the data they provide should be treated as preliminary. A total of 223 articles were scrutinized, including three conference abstracts, two book chapters, and one website with medical reports.

The figure drafts were created by the first author in collaboration with a graphic designer, Anna Lisiecka, using Affinity Designer software version 1.10.6. Senior authors revised them. The artificial intelligence programs ChatGPT Images 2.0 and Gemini Imagen v4.0 were used on 14 May 2026, 10 July 2026, 28 August 2026, and 1 September 2026 for the final processing of the figures, i.e., to achieve better visual and aesthetic effects and a symmetrical arrangement of elements within the figures. These conceptual figures comply with the journal’s AI policy.

## 2. The Biology of Glioblastoma as a Therapeutic Challenge

The limited efficacy of glioblastoma treatment—despite the implementation of advanced diagnostics and multimodal protocols, including experimental strategies—stems, among other factors, from its extraordinarily complex molecular profile. This profile is characterized by dysfunctions in key signaling pathways, including PI3K/AKT/mTOR, Wnt (Phosphoinositide 3-kinase (PI3K)/Protein Kinase B (AKT)/mechanistic target of rapamycin), NF-κB (nuclear factor kappa-light-chain-enhancer of activated B cells), and TGF-β (Transforming growth factor beta), which drive aggressive growth, high invasiveness, and chemoresistance in neoplastic cells [14]. The biologic landscape of glioblastoma is further complicated by intense microvascular proliferation and the presence of ischemic necrotic foci. Rapid neoplastic cell expansion, occurring in parallel with pathological angiogenesis, leads to chronic intratumoral hypoxia. Newly formed blood vessels in glioblastoma (GBM) provide only limited perfusion to the bulk of the tumor, impairing oxygen transport, exacerbating the hypoxic state, and restricting delivery of cytostatic agents to the tumor core [21]. Hypoxia—defined as a state in which the metabolic oxygen demand of cells exceeds their actual availability—is a critical adverse prognostic factor. The phenomenon of hypoxia in glioblastoma exerts a direct influence on both patient prognosis and the efficacy of standard therapeutic modalities, including radiotherapy, chemotherapy, photodynamic therapy, and immunotherapy. It is worth emphasizing that hypoxia is not exclusive to advanced stages of glioblastoma. Oxygen deficits play an equally critical role in the biology of micro-metastases (usually intracranial), early recurrences, and within surgical margins exhibiting microscopic tumor infiltration [22,23].

In response to oxygen deprivation, the protein subunits HIF-1α and HIF-2α (Hypoxia-Inducible Factors) are stabilized in tumor cells. Elevated levels of these proteins in diagnostic biopsies constitute a significant negative prognostic factor, correlating with increased mortality in CNS malignancies, including glioblastoma [24]. Clinical observations are mirrored in experimental studies, in which genetically induced overexpression of HIF-1α stimulates tumor growth, whereas loss of its activity inhibits neoplastic progression [25]. Activated hypoxia-inducible factor 1 (HIF-1α) upregulates, among other targets, vascular endothelial growth factor (VEGF), leading to pathological angiogenesis and, consequently, tumor expansion. HIF also regulates numerous proteins involved in the mechanisms underlying chemoresistance and multidrug resistance (MDR) in glioblastoma [26]. *ABCG2* (ATP-binding cassette sub-family G member 2) of the ABC transporter family, as well as *MGMT* (O^6^-methylguanine-DNA methyltransferase), coding for a DNA repair enzyme, are HIF-1 target genes to which HIF-1 binds at their HREs (hypoxia response elements) [27,28]. Moreover, HIF-1, by binding to SP1 (specificity protein 1) and displacing c-Myc, downregulates MSH2 (MutS homolog 2) and MSH6 of the DNA mismatch repair (MMR) pathway. Although TMZ induces guanine methylation, glioma cells with mismatch repair deficiency can tolerate O6-methylguanine paired with thymine. Thus, deficient mismatch repair is protective towards glioblastoma cells by preventing them from entering endless futile repair cycles that can kill cancerous cells. MMR deficiency plays a significant role in TMZ resistance, while the role of MGMT in TMZ resistance primarily applies to promoter unmethylated cases [29,30,31]. Notably, under hypoxic conditions, the expression of both MGMT and BRCP/ABCG2 (Breast cancer resistance protein/ATP-binding cassette subfamily G member 2), along with HIF-1α, increases in glioblastoma cells [32]. Over-stabilization of HIF-1α reduces malignant glioma cells’ responsiveness to TMZ, including glioblastoma stem-like cells [28,32]. Molecular or hyperoxic targeting of HIF-1 sensitized malignant glioma cells to TMZ [28,32]. It has been postulated that HIF-1α is a master switch for the transition of tumor cells from oxidative to anaerobic glycolysis under hypoxic conditions by regulating the transcription of the glucose transporter GLUT1, hexokinase II (HKII), lactate dehydrogenase A (LDHA), and pyruvate dehydrogenase kinase 1 (PDK1), among others [33]. This phenomenon occurs in the bulk of the tumor and in the mesenchymal subtype of glioma stem cells, creating a hypoxic supply of energy and nucleotides that promotes proliferation and efficient DNA repair. This metabolic state also creates an acidic microenvironment conducive to immunosuppression. Glycolysis contributes significantly to radiotherapy resistance through the enzymatic and non-enzymatic actions of Hexokinase (HK), Pyruvate Kinase M2 (PKM2), Lactate Dehydrogenase A (LDHA), and Enolase (ENO). It also drives chemotherapy resistance by avoiding oxidative phosphorylation (OXPHOS)-related oxidative stress and stimulating NADPH (Nicotinamide adenine dinucleotide phosphate)production via the pentose phosphate pathway (PPP) [34,35].

The critical regulators of HIF are the antisense long non-coding RNAs HIF1A-AS1 (Hypoxia-Inducible Factor 1 Alpha-Antisense RNA 1) and HIF1A-AS2, which augment HIF-1α activity, thereby supporting altered cell metabolism (glycolysis) and enhanced tumor growth [36,37]. In particular, HIF1A-AS2, which partially overlaps HIF1α in an antisense orientation, is activated under hypoxic stress in the tumor microenvironment. It plays a major role in maintaining mesenchymal GSCs (glioma stem cells), a particularly durable and aggressive GSC subpopulation [38]. Mechanistically, HIF1A-AS2 binds to IGF2BP2 (Insulin-like growth factor 2 mRNA-binding protein 2) and DHX9 (DExH-Box Helicase 9), supporting the expression of HMGA1 (High Mobility Group AT-Hook 1), the major mediator of GBM therapy resistance, metabolic reprogramming, tumor progression, cell growth, stemness, and immune evasion. The multifaceted role of HIF1A-AS2 has been further explored using a bioinformatics approach that integrates transcriptomic and clinical data from the TCGA, IvyGAP, GEO, and STRING databases [39,40]. It has emerged as a promising therapeutic target across several investigational studies focusing on metabolic reprogramming, invasiveness, and immune evasion in GBM and other malignant neoplasms [40,41,42,43,44].

Thus, HIFs function as master transcriptional regulators, activating genetic programs responsible for fundamental aspects of tumor biology. They influence progression, invasiveness, and cell survival by inducing genetic instability and fostering resistance to radiotherapy [45]. Therefore, the hypoxia-dependent signaling axis is a crucial target for innovative future targeted strategies to breach glioblastoma’s endogenous defenses.

Also, from the perspectives of radiobiology and pharmacology, glioma stem cells (GSCs) present extreme insensitivity to ionizing radiation and a broad spectrum of cytostatics. This glioblastoma cell subpopulation can initiate tumor formation and multipotency, residing in hypoxic niches that maintain their stemness [32]. Simultaneously, this environment promotes clonal selection of the most virulent cellular subpopulations, inevitably leading to disease progression despite intensified therapeutic efforts [46]. In this regard, HIF-2α is of particular significance in glioblastoma pathogenesis. It has been postulated that, unlike the ubiquitously expressed HIF-1α subunit, which acutely responds to hypoxia, the activity of HIF-2α determines the functionality of GSCs under chronic hypoxia, thereby conditioning their resistance to treatment and their capacity for population regeneration [47]. HIF-2α activity is essential for maintaining the pluripotent phenotype of stem cells, primarily through the direct regulation of transcription factors such as Oct4 (octamer-binding transcription factor 4) [48,49,50]. HIF-2α has been shown to confer resistance to oxidative stress and radiation, among other stressors, by regulating GPx1 (Glutathione Peroxidase 1) [47,51,52]. HIF-2α is also crucial in protecting tumor cells against DNA-targeting and tubulin-binding drugs while promoting cell survival and tumor angiogenesis [53,54]. HIF-2α plays a major role in maintaining the hypoxia-induced stemness of glioblastoma cells [55]. These mechanisms allow the neoplastic population to retain the ability to reconstruct its structure despite ongoing therapy. Experimental silencing of HIF-2α expression leads to a drastic inhibition of cellular division within the GSC population. Consequently, the tumor loses its regenerative capacity, opening avenues for new therapeutic strategies to eradicate the most resistant cellular niches [56].

Intratumoral hypoxia also alters the immunological properties of glioblastoma, causing its reprogramming towards a highly immunosuppressive tumor microenvironment characterized by the recruitment of Tregs, M2 macrophages, and myeloid-derived suppressor cells, as well as the inhibition of antitumor CD8+ T lymphocytes and NK cells. Hypoxia also modifies the profile of neuroinflammation in glioblastoma, as exemplified by elevated levels of secreted pro-inflammatory mediators such as inteleukins IL-1β, IL-6, IL-8, and TNF-α (Tumor Necrosis Factor alpha), along with immunosuppressive mediators including IL-10, CCL22 (Chemokine (C-C motif) ligand 2), and CXCL12 (C-X-C motif chemokine ligand 12) [57,58,59,60,61].

All these mechanisms necessitate intensified research into innovative methods to overcome chemo- and radioresistance induced by specific tumor properties. The unique physicochemical conditions of this biotope—particularly deep tissue hypoxia—form a barrier that drastically impairs the bioavailability and effectiveness of classical treatment protocols.

## 3. Hyperbaric Oxygen Properties Utilized in Neurooncological Combination Therapies

The standard clinical indications for therapy with hyperbaric oxygen (HBO) include the management of decompression sickness, carbon monoxide poisoning, treatment of thermal burns, and the management of non-healing wounds [62]. However, due to its properties, HBO is also being considered an adjuvant treatment in neurooncology.

By definition, HBO treatment exposes the patient to the inhalation of 100% oxygen at an ambient pressure determined by values exceeding 1.0 atmosphere absolute (ATA)—standard baric parameters recorded at sea level (1013.25 hPa). Such a reconfiguration of physical parameters forces a rapid progression of partial oxygen pressure within the patient’s system [63].

Consequently, the implementation of Henry’s Law in medical practice results in a surge in gas solubility in the plasma, rendering tissue oxygenation independent of hemoglobin saturation levels. This mechanism enables the efficient diffusion of oxygen molecules to distal hypoxic tumor niches. Using high-oxygen mixtures allows concentrations of this element in tissue structures to reach several times normal levels. This mechanism enables the successful breaching of the hypoxia barrier, particularly within insufficiently vascularized tumor beds and regions affected by ischemia.

Therefore, HBO modifies the tumor microenvironment by markedly improving neoplastic tissue oxygenation, thereby facilitating conventional therapeutic regimens, such as radiotherapy, chemotherapy, and photodynamic therapy (PDT), which require oxygen to kill tumor cells or enhance their antiglioma effects [64]. The application of HBO for combination therapies is summarized in Figure 1.

However, a key aspect of HBO’s impact is, above all, the impairment of the tumor’s regenerative mechanisms by targeting the subpopulation of cancer stem cells (CSCs). The application of HBO (1.5 atm for 1.5 h/d) markedly reduces GBM cells’ ability to form neurosphere-like structures. This phenomenon, observed during primary and secondary neurosphere formation assays, provides evidence of inhibition of processes responsible for tumor expansion and self-renewal. Furthermore, reduced levels of stemness markers, including CD133 (Cluster of Differentiation 133), OCT4 (octamer-binding transcription factor 4), and SOX2 (SRY-box transcription factor 2), were observed. Glioblastoma stemness was found to be suppressed in both in vitro and in vivo models. GSCs are crucial in tumor renewal, suggesting that HBO combined with conventional treatments may improve therapeutic outcomes [71]. The above data indicate that HBO not only inhibits the development of CSCs but, above all, serves a sensitizing function by eliminating the hypoxic state that favors HIF-1α expression. This allows for the formulation of a precise research hypothesis: HBO therapy attenuates the regenerative potential of GBM stem cells (their “disarming”) without causing their immediate mechanical destruction. These results suggest that the primary function of HBO is to “unmask” stem cells to the effects of chemotherapy and radiotherapy by stripping them of the protection provided by the hypoxic microenvironment [71].

The recent report shows that HIF-1α and HIF-2α influence the formation of glioblastoma cells in both in vitro and in vivo settings. One research group used dual HIF-1α and HIF-2α knockouts in glioblastoma cells and found that dual-knockout cells exhibit the highest apoptosis rate but the lowest levels of stemness and invasion. Lower expression of stemness markers (CD133 and Nestin), VEGF, and transcription factors (Sox2, Oct4, and Klf4- Krüppel-like factor 4) was detected in dual-knockout glioblastoma cells. Likewise, HBO alone (2.5 ATA) decreased the expression of HIF-1α and HIF-2α, as well as CD133, Nestin, VEGF, Oct4, and Klf4, in unmodified tumor cells. However, after TMZ exposure, HBO-treated cells exhibited increased apoptosis rates and LDH release. At the same time, tumor size and weight were reduced in mice, resulting in increased survival compared with the TMZ-only group. This suggests that targeting both HIF-1α and HIF-2α may in part underlie the synergistic effect of the HBO and chemotherapy approach. Other reports also emphasize the importance of dual targeting of HIF-1α/HIF-2α to achieve the highest rate of tumor cell death and note the compensatory increase in HIF-1α expression in HIF-2α-knockout cells [74]. The study also suggests that HBO can be used only as adjuvant therapy to potentiate the beneficial effects of chemotherapy, as HBO used alone tended to increase tumor size and reduce mouse survival [70]. In another study, ATP-binding cassette sub-family G member 2 (ABCG2), a tumor stem cell marker and drug efflux transporter, was shown to be upregulated by HIF-1α and HIF-2α. The experiment was conducted in vitro and in vivo and showed that ABCG2, HIF-1α, and HIF-2α exhibit lower expression after HBO treatment. The expression of CD133 and CD15, stem cell marker proteins, was significantly lower in the dual HIF1α/HIF2α knockout than in the single HIF1α or HIF2α knockouts. ABCG2 is responsible for multidrug resistance in cancer cells, making it another potential therapeutic target. Thus, although HBO alone might support tumor proliferation, when combined with TMZ, it suppresses stemness more than TMZ alone and increases chemosensitivity [75].

A possible explanation could be that HBO alone, on a post-treatment rebound, increases tumor vascularity and proliferation, which are unopposed after a chemotherapeutic agent is withdrawn [76]. The same phenomenon, though, tends to increase the tumor’s vulnerability by exposing dividing cells and facilitating the distribution of chemotherapy agents through the improved tumor vascular network. As mentioned elsewhere in the text, the very mechanism of sensitization may, in part, rely on an increased supply of oxygen as a substrate for chemotherapy-induced cell death mechanisms. However, these results are somewhat at odds with other reports that reported either no effect of HBO alone on cell number or a minor suppressive effect on cell viability. One group found that HBO alone at 2 ATA did not affect cell number, but a small but significant decrease in malignant glioma cell viability after 24 h of incubation (T98G cells) (American Type Culture Collection, Manassas, VA, USA) [72]. In contrast, others reported no effect on the proliferation of C6 glioma cells (Type Culture Collection of the Chinese Academy of Sciences, Shanghai, China) (at 2.5 ATA) [77] or reduced growth of BT4C (Brain Tumor C4) rat glioma xenografts (2 bar) [78]. Moreover, in a glioma mouse model using GBM8401 cells, repeated HBO therapy alone at 1.5 ATA had no effect on tumor growth and did not significantly affect the proliferation of GBM cells (GBM8401 or T98G; Bioresource Collection and Research Center, Hsinchu City, Taiwan and American Type Culture Collection, Manassas, VA, USA) in vitro [71]. Therefore, adjusting HBO dosing seems crucial to avoid excessive HBO regimens commonly used in research studies. As noted by several authors, HBO parameters for neurooncological patients ought to be kept closer to the lower boundary of the therapeutic range [79,80,81]. Both modified drug formulation and avoidance of excessive hyperbaria possess the potential to reduce the risk of unwanted HBO—drug interactions. Observing these principles allowed researchers to combine HBO with doxorubicin-loaded liposomes (Doxil), as doxorubicin is currently contraindicated for use with HBO due to a risk of cardiotoxicity, thereby synergistically inhibiting H22 tumor growth by over 91% [82].

Research has also shown that HBO targets macrophages that can be active in a classical (M1) or alternative (M2) manner. M1 macrophages act in a pro-inflammatory and tumor-killing manner, while M2 macrophages promote an anti-inflammatory response and angiogenesis. Tumor-associated macrophages (TAMs) are polarized toward the M2 phenotype, thereby supporting cancer cell survival. Scientists tested the influence of HBO on macrophage phenotype modulation toward the M1 state. Both in vitro and in vivo assays confirmed that HBO shifts macrophage phenotype from M2 to tumoricidal M1. It downregulates HIF signaling and shifts the local cytokine balance, reducing anti-inflammatory mediators such as IL-10 while increasing pro-inflammatory signals such as TNF-α and IL-1β. Additionally, increased reactive oxygen species (ROS) levels stimulate inflammatory pathways [83].

The integration of HBO with neuro-oncology therapies could be applied to both protocols for newly diagnosed lesions and cases of neoplastic progression. However, the effect of treatment may not be limited to the direct eradication of tumor biomass; hyperbaric oxygen therapy is expected to play a critical role in mitigating and preventing iatrogenic brain tissue damage. This specifically concerns radiation-induced changes following stereotactic radiosurgery (SRS) [84]. By stimulating repair processes and reducing vasogenic edema, HBO could become an indispensable element of neuroprotective coverage, thereby improving patients’ quality of life and expanding the safe range for aggressive local treatments [85]. HBO protocols reviewed in the manuscript for combination therapies with primary treatments for glioblastoma and selected other tumor types are presented in the summary Table 1.

## 4. HBO and Radiotherapy

In the vast majority of analyzed cohorts (including studies focused on head and neck neoplasms), HBO was confirmed to be a highly effective radiosensitizer, significantly improving local control of the primary tumor. The majority of studies indicated a neutral or beneficial effect of hyperbaric exposure on overall survival and a reduction in the risk of metastatic spread, with no evidence of disease progression or high-grade radiation necrosis [85,86,87,88,90,91]. Recent data reaffirm the status of HBO as a safe and highly desirable adjuvant intervention, particularly in mitigating radiation-induced complications and post-operative complications in patients with solid tumors. Despite highly promising findings, authors of recent studies emphasize the need to continue prospective clinical trials in expanded patient cohorts to enable even more precise calibration of HBO’s therapeutic role [97].

In contemporary radiation oncology, external beam radiation therapy (EBRT) is a cornerstone of the therapeutic strategy for glioblastoma [98]. The systematic evolution of radiation technologies and the implementation of advanced Image-Guided Radiation Therapy (IGRT) systems have led to a radical optimization of dose deposition precision within the target volume. The current standard of care is based on a combined paradigm in which the concomitant use of fractionated irradiation and chemotherapy generates mutually reinforcing biological effects, thereby significantly increasing local control of the neoplastic process [99].

To maximize radiation efficiency, HBO increases oxygen supply within the tumor biomass, thereby substantially augmenting the genotoxic impact of ionizing radiation by potentiating free-radical-induced DNA damage [100]. Oxygen increases radiosensitivity predominantly with low linear-energy-transfer (LET) radiation, which remains a cornerstone of radiotherapy for gliomas [101]. However, even in largely investigational approaches using high-LET radiation, which is much less oxygen-dependent, therapeutic effectiveness remains somewhat oxygen-dependent due to LET heterogeneity within the radiation beam and the presence of extremely hypoxic tumor regions [102,103].

Interestingly, both low-LET radiation with HBO and high-LET radiation have been proposed as approaches to combat radioresistance in glioma stem cells to radiotherapy [104,105,106,107,108].

As mentioned before, HBO may act as a mitigating agent, promoting regeneration and limiting radiation necrosis within the healthy brain parenchyma. Thus, by elevating the partial pressure of oxygen within the tumor biomass, HBO drastically increases the susceptibility of neoplastic cells to ionizing radiation, thereby enabling much more efficient elimination of the disease focus while sparing surrounding structures.

However, it must be noted that the efficacy of this strategy is highly variable among individuals and remains closely correlated with the lesion’s histopathological features, molecular profile, and primary anatomical topography [69].

## 5. HBO and Synthetic Antiglioma Chemotherapeutic Agents

The pharmacological activity of numerous cytostatics is fundamentally based on the generation of Reactive Oxygen Species (ROS). These highly reactive molecules arise within eukaryotic cells as a response to oxidative stress induced by fluctuations in oxygen availability, ionizing radiation, or exposure to toxic xenobiotics. While ROS participate in cell signaling under physiological conditions, their excessive production stimulates irreversible DNA destabilization, consequently triggering cell death.

In this regard, hyperbaric oxygen (HBO) emerges as a valuable supportive component. By providing excess oxygen molecules, it intensifies the oxidative stress induced by cytostatic administration, acting as a biological amplifier of the cytotoxic effect. Additionally, hyperbaric oxygen, by ameliorating inefficient tissue perfusion, enhances the distribution of cytotoxic agents within the tumor microenvironment. Such optimization of intracellular drug transport can significantly increase chemosensitivity, paving the way for more effective eradication of the tumor cells [69,109].

Clinical evidence, particularly from studies on carboplatin-based protocols, clearly indicates that the association of chemotherapy with HBO provides a statistically significant survival advantage for patients struggling with recurrent high-grade gliomas [67].

Clinical analysis and in vitro studies confirm the measurable, beneficial impact of HBO on the efficacy of combating diverse oncological entities, especially in models that combine hyperbaria with cytostatics derived from chemical synthesis. It has been observed, among other findings, that exposure to high partial pressure of oxygen significantly enhances the antiproliferative potential of melphalan and gemcitabine, resulting in more effective eradication of breast epithelial cells [110].

However, the cornerstone of glioblastoma chemotherapy is temozolomide (TMZ), which, in the body, is converted to its active form, monomethyl triazenoimidazole carboxamide (MTIC). Then, it methylates the DNA, causing damage and leading to tumor cell death. When the repair enzyme O^6^-methylguanine-DNA methyltransferase (MGMT) is highly expressed in glioma cells, it removes methyl groups, conferring drug resistance and rendering treatment ineffective [29,111]. In this context, the complementary combination of hyperbaric oxygenation with temozolomide (TMZ)-based chemotherapy represents an exceptionally promising therapeutic strategy, striking at the very foundations of neoplastic resistance. As discussed earlier, HBO targets HIF-dependent hypoxic adaptation within the tumor and its downstream effectors that form molecular barriers to TMZ action. However, further studies are needed to delineate its detailed impact, e.g., on MGMT. Moreover, across a spectrum of diseases, HBO increases drug bioavailability in the poorly vascularized core of the lesion. Additionally, it promotes the generation of reactive oxygen species (ROS) which, together with TMZ metabolites, lead to neoplastic cell death. To increase drug bioavailability, HBO can act by alleviating HIF-1-dependent collagen synthesis and drug efflux pumps, reducing tissue edema, and inducing vascular normalization [73,112,113]. The synergy between TMZ metabolites and ROS, the latter producing 8-oxoguanine lesions, increases the overall burden of alkylated and oxidized DNA. Damage to mtDNA (mitochondrial DNA) and cellular respiration, along with ROS-induced depletion of GSH cellular pools, further potentiates therapeutic oxidative stress [114,115,116]. In addition, recent studies have shown that apart from the cytotoxic methyldiazonium cation, another major TMZ secondary metabolite (the primary being MTIC, chemically known as 5-(3-methyltriazen-1-yl)imidazole-4-carboxamide)-5-aminoimidazole-4-carboxamide (AIC) may act as a precursor in purine biosynthesis, thereby aiding in rebuilding tumor energy reserves and contributing to chemo- and radiation- resistance [117]. AIC and its intracellular derivatives have been implicated in the upregulation of antioxidant defenses [118]. Although this requires scientific verification, HBO, by contributing excess ROS, might oppose the antioxidant action of AIC, overwhelm purine biosynthesis and purine salvage pathways, and thereby, to a degree, compensate for this inherent drawback of TMZ therapy. Nevertheless, it has been demonstrated that the forced increase in oxygen partial pressure within the tumor microenvironment enhances the cytotoxic potential of TMZ by effectively neutralizing intratumoral hypoxic niches and decreasing tumor stemness [96]. This mechanism is pivotal, as tissue hypoxia induces the expression of DNA repair proteins that directly counteract the alkylating action of temozolomide [119]. In vitro analyses using a human glioblastoma cell line (U251, KeyGen Biotech, Nanjing, China) provided evidence of a beneficial therapeutic interaction between hyperbaric oxygen and temozolomide [65]. Compared with monotherapy (TMZ alone or HBO alone), the combined protocol showed markedly greater effectiveness in inhibiting proliferative potential and inducing programmed cell death (apoptosis) in neoplastic cells. A statistically significant decrease in vascular endothelial growth factor (VEGF) levels was also observed, suggesting inhibition of the tumor’s angiogenic potential under optimized oxygenation. Simultaneously, the combination therapy reduced MRP-1 (Multidrug Resistance-associated Protein 1) protein expression, which is responsible for the active transport of drugs out of the cell. The reduction in this protein level constitutes a mechanism for overcoming multidrug resistance in GBM, directly increasing the intracellular bioavailability of cytostatic agents and rendering glioma cells more susceptible to the lethal effects of temozolomide [65]. It has also been experimentally demonstrated that even normobaric hyperoxia could, to some extent, enhance the sensitivity of chemosensitive and chemoresistant glioblastoma cells to TMZ in vitro [120]. However, studies have shown that HBO provides a clear advantage in oxygenating deep tumor structures compared with hyperoxia alone [106,121].

Convergent conclusions were reached by other researchers, who confirmed that HBO has significant potential to attenuate glioma cell proliferation and limit inflammatory infiltrates within the neoplastic microenvironment. These authors demonstrated that HBO serves as an effective sensitizing agent for the action of nimustine (ACNU), a structural analog of lomustine [94,122,123]. The mechanism of this phenomenon centers on the rapid increase in oxygen partial pressure within tumor structures, which suppresses the hypoxia adaptation signaling pathway by markedly reducing the expression of the HIF-1α factor. The loss of this molecular axis leads to a cascade of decreases in proangiogenic mediators, such as VEGF and matrix metalloproteinase-9 (MMP-9), which are key determinants of tumor invasiveness and migratory capacity. Furthermore, optimizing tissue oxygenation inhibits the expression of key pro-inflammatory and anti-apoptotic factors, including TNF-α, IL-1β, and the NF-κB complex. Such a broad spectrum of genomic and proteomic effects in neoplastic cells confirms that hyperbaric oxygen therapy not only mechanically eliminates hypoxia but also actively reprograms the tumor phenotype, thereby making it more susceptible to cytotoxic pharmacological intervention and limiting its potential to reconstruct inflammatory niches that favor progression [94].

## 6. HBO and New Chemical Syntheses for Investigational Drugs

As noted above, HBO has been shown to enhance the antiglioma efficacy of TMZ, a crucial component of glioblastoma therapy under the Stupp protocol. Nevertheless, temozolomide does not work in every case and oftentimes is followed by tumor recurrence. Therefore, there is a need to explore new drug classes to improve treatment efficacy. Amphiphilic isothiourea derivatives represent a new class of promising antiglioma agents. Due to the presence of a highly basic functional group, these molecules undergo protonation at physiological pH. This cationic form dictates their biological profile. Furthermore, the solid-state isothiourea salts exhibit enhanced aqueous solubility compared to the reaction substrates. Within this group of compounds, S-benzyl derivatives (ZKKs) are of key importance, exhibiting in vitro cytotoxic activity and the ability to induce apoptosis. This activity has been documented against glioma, prostate cancer, and leukemia cell lines. According to studies by Kamińska et al. [124], the presence of an N-substituted alkyl or allyl group (ZKK-2, -3, -4, and -5) determines the highest antitumor activity of these compounds. Conversely, cytotoxicity was reduced by attaching a bulky N-phenyl substituent (ZKK-6) or by substituting the bromine atom in the aromatic ring with chlorine or fluorine (ZKK-7, -8) [124,125,126,127].

Further structural modifications led to derivatives with higher biological activity. Ultimately, among all tested substances, compounds ZKK-2, ZKK-3, ZKK-9, and ZKK-13 exhibited the highest anticancer efficacy. These analyses led to the identification of derivatives with the most potent cytotoxic activity against glial-derived tumor cells. Among them, compound ZKK-13, bearing two N-isopropyl groups, proved to be the most effective inhibitor of cell proliferation and viability in the T98G and SEGA cell lines (American Type Culture Collection, Manassas, VA, USA) [128].

Despite the expected CK2 inhibitory potential arising from structural analogy to known inhibitors of this kinase, screening against a panel of 130 protein kinases revealed very weak activity of compound ZKK-3 (N, N’-dimethyl-S-(2,3,4,5,6-pentabromobenzyl)isothiourea) against this enzyme. Conversely, it demonstrated the capacity to specifically inhibit other kinases (above 70%), including PKD1 (Protein Kinase D1_, ERK8 (Extracellular signal-regulated kinase 8), NEK2A (Never In Mitosis A-related kinase 2, isoform A), PIM1 (Pim-1 proto-oncogene, serine/threonine kinase), PIM3, IGF-1R (Insulin-like growth factor 1 receptor), and IR (Insulin Receptor). [126]. For instance, the compound N, N’-dimethyl-S-(2,3,4,5,6-pentabromobenzyl)-isothiouronium bromide (ZKK-3) has been reported to suppress protein kinase D1 (PDK1), thereby thwarting glioblastoma cells’ protection against oxidative stress, according to an in vitro study. Analyses showed that this derivative statistically significantly reduces the viability and proliferative potential of glioblastoma cells in vitro. Interestingly, ZKK-3’s cytotoxicity significantly increased under hyperbaric conditions [72]. A question arises as to whether novel synthesis, such as the ZKK line, may serve as a substitute for TMZ. For one, both TMZ and ZKK3 are suitable for combination therapies, including those with HBO. However, ZKK1 MW is 642 Da while ZKK3 is 670 Da, far more than TMZ; hence, their bioavailability after oral administration would pose a hindrance. Therefore, several strategies can be employed to achieve controlled delivery of ZKK to the central nervous system. Here, ultrasound-induced opening of the BBB is focused on the tumor-affected regions. Second, exosomes laden with ZKK (including Human Serum Albumin (HSA)-bound ZKK to target albumin-binding receptors on glioblastoma, e.g., SPARC) could be of help, delivering ZKK cargo via the transcytosis pathway [129,130,131,132]. Last but not least, HBO could facilitate BBB crossing of molecules heavier than 500 Da, and it has already been postulated that this modality has the potential to be used therapeutically for controlled drug delivery through the BBB [133]. HBO could also increase EV passage into the brain, as it has been shown to increase transcytosis in the non-ischemic brain [134]. The molecular mass of Evans blue is 960.8 Da; however, when bound to plasma albumin, the resulting complex that crosses the BBB under HBO conditions has a mass of about 66 kDa. HRP (horseradish peroxidase), with a molecular mass of 44 kDa, passed the BBB under hyperbaric air. This suggests that HBO parameters for enhanced delivery of the HSA-bound and HSA-unbound fractions of ZKK can be adjusted to balance BBB opening with unwanted effects, including oxygen toxicity.

In the case of ZKKs, further studies of the effect of ZKKs on the alkylation processes, linked to an array of oncostatic medications, as well as their effect on the enzymes involved in the chemical basis of antiglioma action of TMZ, including in silico redesign towards attaining such properties, are warranted [135,136]. It is known that thioureas inhibit Lysine-Specific Demethylase 1 (KDM1A/LSD1), which has recently been implicated in cancer resistance through multiple mechanisms, including increasing hypoxia-inducible factor-1a (HIF-1a)-mediated transcriptional activation via its demethylation [137,138,139]. In particular, LSD1, highly expressed in GBM, plays a major role in GBM resistance to TMZ [140]. The inhibition of KDM1A/LSD1 augments the efficacy of TMZ in reducing glioblastoma stem cell viability and neurosphere-forming capacity, in part by attenuating DNA DSB repair pathways [141,142]. Of note, KDM5B (JARID1B), also elevated in glioblastoma, is upregulated by HIF-1α and contributes to mechanisms of TMZ (Sigma-Aldrich, St. Louis, MO, USA) resistance in glioblastoma cells from patients [143,144]. Thus, ZKK and its derivatives may significantly enhance TMZ’s tumoricidal activity, especially when combined with HBO. Figure 2 illustrates established and putative mechanisms underlying the antiglioma effects of ZKKs in combination with TMZ and HBO.

## 7. HBO and Selected Antiglioma Phytopharmaceuticals and Nutraceuticals

A promising research direction involves using HBO as a catalyst for natural substances. Compounds derived directly from the plant kingdom currently enjoy sustained scientific interest due to their unique biological activity and relatively low systemic toxicity profile. A perfect example of such cooperation is the combination of oxygen therapy with paclitaxel, a key representative of the taxane group, originally isolated from the bark of the Pacific yew (*Taxus brevifolia*). It has been shown that HBO enhanced the paclitaxel-induced inhibition of mitotic divisions in cancer cells [110].

Identifying substances that, when combined with the standard therapeutic protocol (radiotherapy and temozolomide-based chemotherapy), exhibit additive or synergistic effects in inhibiting glioblastoma tumor cell proliferation is of paramount importance. Scientific literature points to compounds with proven oxidative potential, among which artemisinin plays a key role. The breakthrough significance of this substance was officially recognized in 2015 with the Nobel Prize in Physiology or Medicine.

Originally isolated in 1971 from *Artemisia annua* L., artemisinin is a sesquiterpene lactone [147]. Its characteristic feature is a unique endoperoxide bridge, which determines its high biological activity. While these compounds are widely used in treating malaria, modern oncology sees immense therapeutic potential in them. Artemisinins have been tested in laboratory investigations and clinical trials across a broad range of malignancies, revealing their effects on cell death, cell cycle arrest, and anti-angiogenic effects [148,149]. The role of hyperbaric oxygenation has also been documented in enhancing the cytotoxic effect of artemisinin in human leukemia cells [93]. However, due to the low solubility of natural artemisinin, numerous derivatives with improved pharmacokinetics have been synthesized, including dihydroartemisinin (DHA). In glioma, these compounds act through a broad spectrum of mechanisms beyond classical apoptosis [150,151].

DHA occupies a special place in research, exhibiting potent action against glioblastoma. Its advantages include high selectivity, limited toxicity to healthy tissues during short-term treatment, and a low potential to generate drug resistance.

Artemisinin and its derivatives show toxicity in animal studies due to prolonged drug availability but remain relatively safe in human clinical use, where artesunate displays the safest profile. Several authors have confirmed that the toxicity of artesunate observed in animal models is predominantly seen with delayed-release intramuscular injections but is rare or absent in standard human treatments [152,153,154]. DHA has been shown to kill drug-resistant glioblastoma tumor cells selectively and to reduce spheroid formation, viability, and tumor growth in a mouse model when combined with the porphyrin enhancer 5-ALA [155]. A fundamental pillar of DHA’s antiglioma activity is the induction of ferroptosis, driven by the accumulation of lipid reactive oxygen species (ROS). Molecular analysis has identified GPX4 (glutathione peroxidase 4) as the primary target; its rapid decline triggers glioblastoma cell death under the influence of DHA [156]. The introduction of HBO into the DHA protocol was investigated to reinforce the therapy. In an environment saturated with high-pressure oxygen, the activity of the DHA endoperoxide bridge is multiplied, leading to an “oxidative burst.” Since DHA simultaneously impairs the cell’s defense system (by reducing GPX4), cancer cells lose the ability to neutralize excess free radicals, resulting in rapid, selective destruction of tumor tissue through intensified ferroptosis [156].

Interesting approaches also include anticancer combination therapies with HBO and the investigational use of nutraceuticals, which are naturally occurring in foods and believed to have medical or health benefits [157]. Among these is ascorbic acid (AA), which at high doses can act as a pro-oxidant, generating hydrogen peroxide that kills tumor cells. An in vitro approach yielded promising preliminary results indicating the interaction between HBO and AA in this respect, showing that AA mediates VM-M3 (VM-M3 murine brain tumor) cell death and that HBO significantly enhances AA’s cytotoxic effect [95]. Theoretical background is further supported by several reports indicating that glioblastoma cells accumulate AA, among other compounds that could work synergistically with HBO to generate therapeutic oxidative stress within the tumor [158]. To this end, glioma-attracted carriers laden with prooxidants or pharmacological agents that synergize with HBO could also be utilized, including EVs.

A promising natural compound that appears suitable for HBO-combined anticancer therapies is curcumin; however, such synergy has been investigated only rarely to date. Noticeably, though, treatment of a female patient suffering from myeloma with high-dose (8 g/day) curcumin alongside weekly HBO resulted in the absence of disease progression prolonged over 5 years [89]; notably, though, even 8 g/day may not guarantee bioavailability. As curcumin is not water-soluble, increasing its bioavailability is of utmost importance, and specialized formulations, such as micellar or nano-scaled liposomal delivery systems, should be employed in future studies [159]. On the other hand, curcumin, as a component of other anticancer combination therapies, has already accumulated a substantial body of literature [160,161]. Curcumin is a natural polyphenol that was first isolated in 1815 by Vogel and Pelletier from the rhizomes of *Curcuma longa* belonging to the ginger family (*Zingiberaceae*). Although curcumin has long been used to treat wounds, respiratory and digestive ailments, it exhibits a broad spectrum of biological activity in clinical and experimental oncology. This compound also has a documented ability to modulate key signaling pathways involved in glioma development and progression [162]. Therapeutic actions of curcumin against gliomas are depicted in Figure 3.

Curcumin is a key modulator of redox homeostasis, exerting a decisive influence on the severity of oxidative stress in tumor tissue. This substance is widely recognized as a factor stimulating the generation of reactive oxygen species (ROS) during pharmacologically induced apoptosis, as confirmed by research reports on glioblastoma and other neoplastic entities [172,173]. Analyses of glioma have demonstrated that simultaneous administration of curcumin and temozolomide (TMZ) leads to a drastic increase in reactive oxygen species (ROS) production in U87MG cell lines, suggesting that curcumin acts as a sensitizer, rendering resistant glioma cells more susceptible to TMZ cytotoxicity. This combined effect on prooxidant activity has been documented in both in vitro and in vivo models. Of note, curcumin also exerts genotoxic activity, inducing DNA damage, which presumably contributes to the anticancer effect. As demonstrated by Beltzig, L et al. [159], in various cell lines, after curcumin removal, the genotoxic effects disappeared, indicating effective and complete repair of DNA damage. Hence, it is of great importance to determine the appropriate timing of curcumin treatment in cancer [159]. Furthermore, the combined administration of the two compounds has been shown to effectively suppress signaling pathways by inhibiting phosphorylation of AKT and mTOR. Furthermore, cell cycle arrest has been demonstrated in specific phases, preventing tumor regeneration and further growth [165]. In addition, curcumin demonstrates significant antitumor activity against glioma by modulating the JAK/STAT pathway, both in vitro and in vivo [163].

In addition to the above mechanisms, the therapeutic potential of curcumin in the treatment of GBM is also manifested through precise epigenetic modulation, including stimulation of the expression of specific microRNAs (miRNAs), such as miR-223-3p, miR-133a-3p, miR-181a-5p, miR-34a-5p, miR-30c-5p, and miR-1290. As indicated by research reports (e.g., Bonafé et al. 2022 [169]), this selective regulation significantly impairs the Akt pathway by directly disrupting its signaling cascade, resulting in increased apoptosis and reduced cancer cell proliferation. Particular importance is attributed to the overexpression of miR-223-3p and miR-133a-3p, which exhibit a bidirectional effect: on the one hand, they inhibit the Akt axis, and on the other hand, they suppress the activity of the transcription factor NF-κB. This multilevel regulation of survival pathways through the modulatory functions of microRNAs is a key element of the argument for using curcumin as an innovative adjuvant in complex oncology strategies [169].

Furthermore, curcumin promotes radiation-induced immunogenic cell death (ICD), leading to increased apoptosis and immune response, as evidenced by increased exposure of calreticulin and the release of heat shock protein HSP70 and ATP (adenosine triphosphate) [174].

A key aspect of curcumin’s action is its role as a promising radiosensitizer and a supportive agent in glioma radiotherapy. This compound has the unique ability to selectively treat different cell types: it acts as a radioprotector, protecting healthy neurons from damage while simultaneously making cancer cells significantly more susceptible to radiation. This dual action offers the potential to increase treatment effectiveness while reducing bothersome side effects. Studies combining curcumin administration with radiation confirm that this combination yields significantly improved results [166].

The radiosensitizing effect of curcumin is primarily due to its ability to modulate key molecular pathways, including the mTOR pathway, which plays a key role in the induction of autophagy. By activating autophagy, curcumin increases the susceptibility of cancer cells to radiation-induced damage, thereby enhancing treatment efficacy. Furthermore, curcumin targets glioma stem cells, often responsible for therapeutic resistance and tumor recurrence. By suppressing the tumorigenic properties of GSCs by inducing autophagy, curcumin not only increases radiosensitivity but also reduces the risk of recurrence [171].

Curcumin enhances the generation of reactive oxygen species, enhancing the cytotoxic effects of radiation and leading to increased apoptosis and reduced cell invasion. Studies have shown that curcumin enhances the efficacy of radiotherapy under both high- and low-linear-energy-transfer conditions in vitro. When combined with radiation, curcumin increases the sub-G1 cell population, elevates reactive oxygen species levels, and ultimately increases apoptosis in glioma cells. These effects are more pronounced with neutron radiation (high LET) than with γ radiation (low LET). Furthermore, the combination of curcumin and neutron radiation significantly inhibits glioma cell invasion, exceeding the effects of either treatment alone or curcumin combined with γ radiation. This increased radiosensitivity, particularly with high-LET radiation, suggests the potential clinical use of curcumin as part of a combination therapy to improve outcomes in glioma patients [175].

Combining curcumin with radiation exposure induces cell cycle arrest in the G2/M phase, which constitutes a significant barrier to further proliferation of glioma cells. A research team led by Zoi and colleagues analyzed the potential of curcumin (Sigma Aldrich, St. Louis, MO, USA) as a radiosensitizing agent. In the experiment, cancer cells were incubated with curcumin and then exposed to radiation doses of 2 Gy or 4 Gy. The effectiveness of this hybrid strategy was compared with the effects of supplementation alone and irradiation alone, assessing parameters of viability, proliferation, and cell cycle progression. The results clearly confirmed that the combined action of curcumin and radiation drastically reduced cell survival in both analyzed lines, exceeding the effects of monotherapy. Furthermore, the combination approach induced a significantly stronger G2/M phase cycle block. Attempts to combine curcumin with temozolomide also yielded interesting findings, resulting in increased cancer cell toxicity. This study demonstrated that curcumin, administered at low doses, exhibits a strong synergistic antiproliferative effect with radiation in vitro. This represents a promising direction for designing new therapeutic regimens, although a full understanding of the underlying molecular mechanisms requires further, in-depth scientific validation [167].

Ghanbari et al. [176] analyzed the combined effects of curcumin, radiotherapy, and hyperthermia using a glioma spheroid model. They hypothesized that curcumin—valued for its anti-inflammatory and antioxidant properties—also stimulates the effectiveness of radiation and high temperature, leading to optimized therapeutic outcomes. The data obtained confirmed a significant effect of this triple combination on the structure of tumor spheroids. The results suggest that curcumin can sensitize glioma cells to both ionizing radiation and thermal stimuli. This synergistic effect significantly enhances overall therapeutic efficacy, making this strategy highly promising for combating resistant tumor masses [176]. On the other hand, analyses conducted by Sminia et al. [177], focusing on the radiosensitizing potential of curcumin (Sigma Aldrich, St. Louis, MO, USA) against the human glioma cell line U251 (American Type Culture Collection, Manassas, VA, USA), yielded different conclusions. This experiment demonstrated that the compound does not exhibit radiosensitizing properties at concentrations realistically achievable in clinical settings during glioblastoma therapy. According to the reported data, curcumin levels within the tumor are too low to elicit cytotoxic effects. This suggests that, despite promising results in other models, physiological barriers limit the ability to achieve the therapeutic dose necessary to support radiotherapy in the patient’s body [177].

Despite its promising efficacy demonstrated in in vitro studies and preclinical models, its clinical implementation is limited by critical pharmacokinetic barriers. A key determinant is its negligible bioavailability, resulting from its marginal solubility in aqueous media, chemical lability, and extensive systemic biotransformation. Combined with the limited penetration of the blood–brain barrier, these factors prevent therapeutic concentrations from being achieved within the tumor microenvironment. Given these limitations, the paradigm of modern neurooncology is leaning toward advanced active ingredient delivery systems—such as nanocarriers, liposomes, and polymer micelles [178]. Their role is to stabilize the curcumin molecule structurally, protect it from premature enzymatic degradation, and ensure selective accumulation and enhanced penetration into the central nervous system, thereby maximizing its therapeutic benefits [179].

It is worth noting that curcumin derivatives are emerging as highly promising therapeutic agents for glioblastoma, demonstrating enhanced antiproliferative activity at lower doses than the parent compound. This superior efficacy results from their optimized solubility, greater chemical stability, and ability to effectively initiate blockade of the mitotic cycle and programmed cell death in neoplastic cells.

A key advantage of these synthetic analogs is their minimal impact on physiological cell populations, which significantly increases their value as precise tools for targeted therapies. By selectively targeting tumor structures while preserving the integrity of healthy tissue, these substances provide a promising foundation for modern glioblastoma treatment protocols [179].

Studies by Maiti et al. [180] have shown that solid lipid curcumin particles (SLCPs) (Verdure Sciences, Noblesville, IN, USA) enhance curcumin bioavailability and anticancer activity against glioma compared with native curcumin, due to improved solubility and stability. Indeed, curcumin administered as a micellar solution has the highest bioavailability and is effective even when administered orally (e.g., in drinking water) [181]. In vitro studies using 20 μM SLCPs on U-87MG (ATCC, Manassas, VA, USA) and U-251MG (European Collection of Authenticated Cell Cultures, Porton Down, UK) glioma cell lines demonstrated significant cell death and inhibition of proliferation, with enhanced effects observed when combined with berberine (Sigma-Aldrich MERCK, St. Louis, MO, USA). SLCP treatment induced apoptosis, as evidenced by increased DNA fragmentation. Additionally, SLCPs disrupted mitochondrial function, leading to decreased mitochondrial membrane potential and ATP levels. Treatment also increased reactive oxygen species production, contributing to oxidative stress and cell death. Importantly, SLCPs inhibited the PI3K/Akt/mTOR signaling pathway, a key pathway for cell growth and survival, with enhanced inhibition observed when combined with berberine. These findings suggest the potential of SLCPs, especially in combination therapies, in the treatment of glioma [180].

Currently, the clinical potential of curcumin is being directly reflected in advanced research projects, as exemplified by the ongoing clinical trial number NCT05768919. It focuses on assessing the therapeutic efficacy of liposomal curcumin, administered in combination with a standard protocol that includes radiotherapy and temozolomide chemotherapy, in patients with high-grade gliomas. Implementing the liposomal formulation in clinical trials directly addresses the aforementioned pharmacokinetic barriers, aiming to leverage curcumin’s molecular action and prooxidant potential to overcome chemoresistance and improve prognosis in this aggressive disease (https://clinicaltrials.gov/study/NCT05768919 (accessed on 5 December 2025)). This trial is also aimed at optimizing the compound’s delivery to intracranial structures.

The potential of curcumin may take on new dynamics when integrated with hyperbaric oxygen therapy (HBO). While HBO reduces tumor hypoxia and generates reactive oxygen species (ROS), curcumin intensifies this oxidative stress within cancer cells, making them even more susceptible to the cytotoxic effects of temozolomide and radiation. Curcumin binds to specific regulatory and metabolic enzymes (such as carbonyl reductases, glutathione S-transferase P1, and NADPH-related enzymes. This results in mitochondrial dysfunction and overproduction of internal ROS [182,183]. Studies across a variety of cell cultures have shown that at 1–5 μM, cells experience a mild, transient increase in intracellular ROS. The definitive threshold at which curcumin switches behavior and prompts cells to produce destructive levels of ROS is 10 μM or higher [184,185,186]. This results in a multi-pronged mechanism of attack: HBO supplies the oxygen necessary for free radical reactions, curcumin destabilizes defense mechanisms and survival pathways (such as PI3K/Akt/mTOR), and standard oncological treatment completes the process of eradicating neoplastic cells. This integrated strategy could maximize therapeutic outcomes while maintaining high protection of healthy neurons, representing one of the most promising directions in modern neurooncology. In addition, as in the artesunate studies, where not the original compound but its derivatives proved suitable for combined therapies, novel curcumin derivatives and analogs should be synthesized and evaluated for suitability in HBO applications. This includes their different formulations and routes of administration. To this end, studying the effect of HBO on the stability of the novel compounds and their vectors is also warranted [82,187]. Figure 4 summarizes the proposed placement of curcumin-based pharmaceuticals amongst standard antiglioma treatment regimens and HBO.

## 8. Safety, Adverse Effects, and Contraindications of Hyperbaric Oxygen Therapy

### 8.1. Adverse Effects

Overall HBO adverse effects can be divided into three parts, of which the most common are different types of barotrauma, followed by consequences of oxygen toxicity and finally, treatment-induced psychological disorders. When air trapped in body cavities fails to equalize with changing pressures, it can stretch or compress tissues, resulting in different types of trauma depending on the air’s location. Ear barotrauma (2–43% of all patients) can manifest as pain, fullness, tinnitus or, albeit rarely (1 in 10,000 treatments), as ear bleeding and eardrum rupture with hearing loss [191]. Sinus barotrauma causes predominantly nasal congestion and painful sensations, rarely epistaxis (1:10,000 treatments). While in the lungs, trapped air may even lead to pneumothorax or air embolism (1 in 50,000 treatments) [192,193]. Dental barotrauma appears least severe, causing tooth squeeze—barodontalgia.

Oxygen Toxicity can pertain to the Central Nervous System (CNS) or cause pulmonary injury and ocular complications. For CNS oxygen toxicity, generalized seizure is a poster child complication, although it occurs in approx. 1:10,000 treatments; it is still the most feared and prevented by many HBO protocols by utilizing air breaks—when patients receive air to breathe typically for 5–10 min each [194]. Pulmonary oxygen toxicity is usually cumulative, as it tends to escalate over prolonged exposures. It develops in part due to insidious symptoms such as a persistent cough and shortness of breath; however, it is largely avoidable in most current HBO applications [192]. However, acute inflammation and edema can be followed by fibrosis and impaired pulmonary mechanical function [195]. Recent studies indicate that the alveolar–capillary barrier is the most sensitive and slowest-recovering part of the lung injury induced by HBO [196].

Ocular side effects of oxygen include hyperoxic myopia (∼0.25 diopters per week), caused by oxygen acting on the crystalline lens (most common). It is very common (in up to 90% of patients); however, it usually gradually resolves within 10 weeks [197]. Cataract formation occurs only in treatment series that greatly exceed 20–60 treatments, whereas over 100 treatments may affect a significant proportion of treated patients, particularly those with existing risk factors [198]. Retinopathy of prematurity affects prematurely born infants and has been discussed elsewhere [199].

Psychological complications associated with hyperbaric oxygen therapy (HBO) comprise claustrophobia and confinement anxiety triggered by being enclosed inside a small space. Patients experience a sudden sense of panic, confinement, and high anxiety. To alleviate the anxiety, medical professionals communicate with patients inside the chamber in real time. Patients may also receive anxiolytic medications or mild sedatives beforehand [81,200]. Some people report fatigue following hyperbaric oxygen therapy, but these findings are inconsistent, as others report elevation in strength and spirit [81,136].

### 8.2. Contraindications and Safety

Amongst contraindications, an untreated pneumothorax represents the only absolute contraindication to hyperbaric oxygenation. Under pressure, a simple pneumothorax can progress into a life-threatening tension pneumothorax. Others are relative contraindications, and these include upper respiratory infections such as chronic sinusitis, as well as emphysema, Chronic obstructive pulmonary disease (COPD), asthma, and tuberculosis. Naturally, HBO is advised against after recent ear, eye, or thoracic surgery or trauma, in high fever (lowers the seizure threshold), and in diabetes HBO can induce hypoglycemia) and in all unstable internal conditions that need medical management first [201].

As for medications, recent therapy with bleomycin (pulmonary toxicity risk) and doxorubicin (cardiotoxicity risk), disulfiram (which increases the risk of oxygen toxicity), and mafenide (Sulfamylon) due to the increased risk of metabolic acidosis and oxygen toxicity [202].

Also, pregnancy creates a relative contraindication, mostly because of the lack of knowledge on how high-pressure oxygen affects a developing fetus. Pregnancy thus is contraindicated unless a critical medical emergency exists [203].

For safety, especially the risk of fire, patients must follow all precautions at HBO facilities. Smoking, possessing flammable materials, or using electronic personal devices are strictly prohibited. Electronic equipment used inside or near a hyperbaric oxygen therapy chamber undergoes strict safety testing and certification [204,205]. Finally, medical professionals operating or entering hyperbaric oxygen therapy chambers must be highly trained, having passed fitness-to-dive physical exams, and hold emergency and hyperbaric medicine certificates. Moreover, the staff must maintain a high level of mental sharpness, emotional stability, and psychological resilience [204,205,206,207].

## 9. Discussion, Conclusions and Future Directions

The foundation of HBO’s effectiveness is its ability to radically reconfigure the tumor microenvironment by eliminating intratumoral hypoxia. This process suppresses the HIF-1α/HIF-2α signaling pathway, resulting in a cascade of decreases in the expression of proangiogenic (VEGF), inflammatory (TNF-α, IL-1β, NF-κB), and invasive (MMP-9; matrix metalloproteinase-9) mediators. By optimizing oxygenation, HBO not only inhibits tumor biomass progression but, above all, restores neoplastic cells’ sensitivity to genotoxic stimuli.

A particularly significant achievement of contemporary research is the demonstration of HBO’s impact on glioma stem cells. Hyperbaric exposure destabilizes the stem phenotype by reducing levels of key pluripotency markers (CD133, OCT4, SOX2). This “biological disarming” impairs their potential for self-renewal and neurosphere formation, rendering them susceptible to standard medical procedures.

New developments in the mechanism of action of combination therapies involving HBO and antiglioma agents appear to provide momentum for clinical studies. In fact, a new study launched this year at Southern Cross University in Australia will test mild HBO (1.35 ATA) as part of active treatment for high-grade gliomas [92]. A recent meta-analysis of 9 RCTs (Randomized Controlled Trials) involving 837 patients found that concurrent HBO significantly improved objective tumor response rates and 3-year overall survival when combined with chemoradiotherapy, supporting the need to advocate for large-scale, multicenter Phase 3 trials [208].

As a complementary method, hyperoxic hyperbaria does not aspire to replace standard medical protocols but radically intensifies their therapeutic potential. It creates a microenvironment that is highly unfavorable to further tumor progression by targeting the adaptive mechanisms that previously protected gliomas from external factors. The seamless integration of these three fundamental pillars—oxygen supplementation, precision irradiation, and targeted cytostatics—opens entirely new perspectives in the fight against the most virulent forms of high-grade gliomas. The anti-glioma pharmaceuticals, which are very effective when tested under normoxic conditions in laboratory settings but encounter a hypoxic tumor microenvironment and the activation of HIF–dependent tumor defenses in clinical practice, are especially suitable for combination therapy with HBO. Another potentially eligible group comprises therapeutics that utilize surplus oxygen to augment their tumor-killing properties, such as catalysts of oxidative cell death or activators of ferroptosis, all of which require further study [209]. Antiglioma agents tend to be most effective in normoxic conditions in vitro, where the continuous activation of OXPHOS genes produces ROS, thereby enabling their tumoricidal action [210]. Upregulation of HIF under hypoxic conditions in tumors may suppress mitochondrial respiratory chain function, thereby hampering this important source of ROS [211,212]. Thus, the addition of HBO for in vivo tumor treatment has the potential to help translate promising laboratory results to oncology clinics. Studies have shown that inhibiting HIF-1 and its downstream targets in cerebral tissues is much more effective under hyperbaric oxygen than under normobaric hyperoxia [213]. It seems that in this promising scenario there is still an oncogene-mediated Warburg effect—the existence of fermenting glucose in tumor cells even when oxygen is abundant—that remains unopposed [214]. This metabolic reprogramming accounts for roughly half of glycolytic flux, with the remainder attributable to HIF-mediated hypoxic adaptation. HBO can thwart hypoxic adaptation; however, whether it can activate the reverse Warburg effect remains to be seen [215]. Definitely, targeting molecularly or pharmacologically altered glucose metabolism (e.g., with Shikonin for PKM2, shRNA for HK2, or inhibitor LMP744 for C-MYC/TOP1- Cellular Myelocytomatosis Oncogene/DNA Topoisomerase 1) is worth considering as an additional combination therapy component to beat glioblastoma [216,217,218,219,220,221,222,223].

Experimental and clinical data position HBO therapy as a promising component of experimental oncologic strategies to improve prognosis and extend survival in patients with HGG who were previously considered treatment-resistant [71].

## Figures and Tables

**Figure 1:**
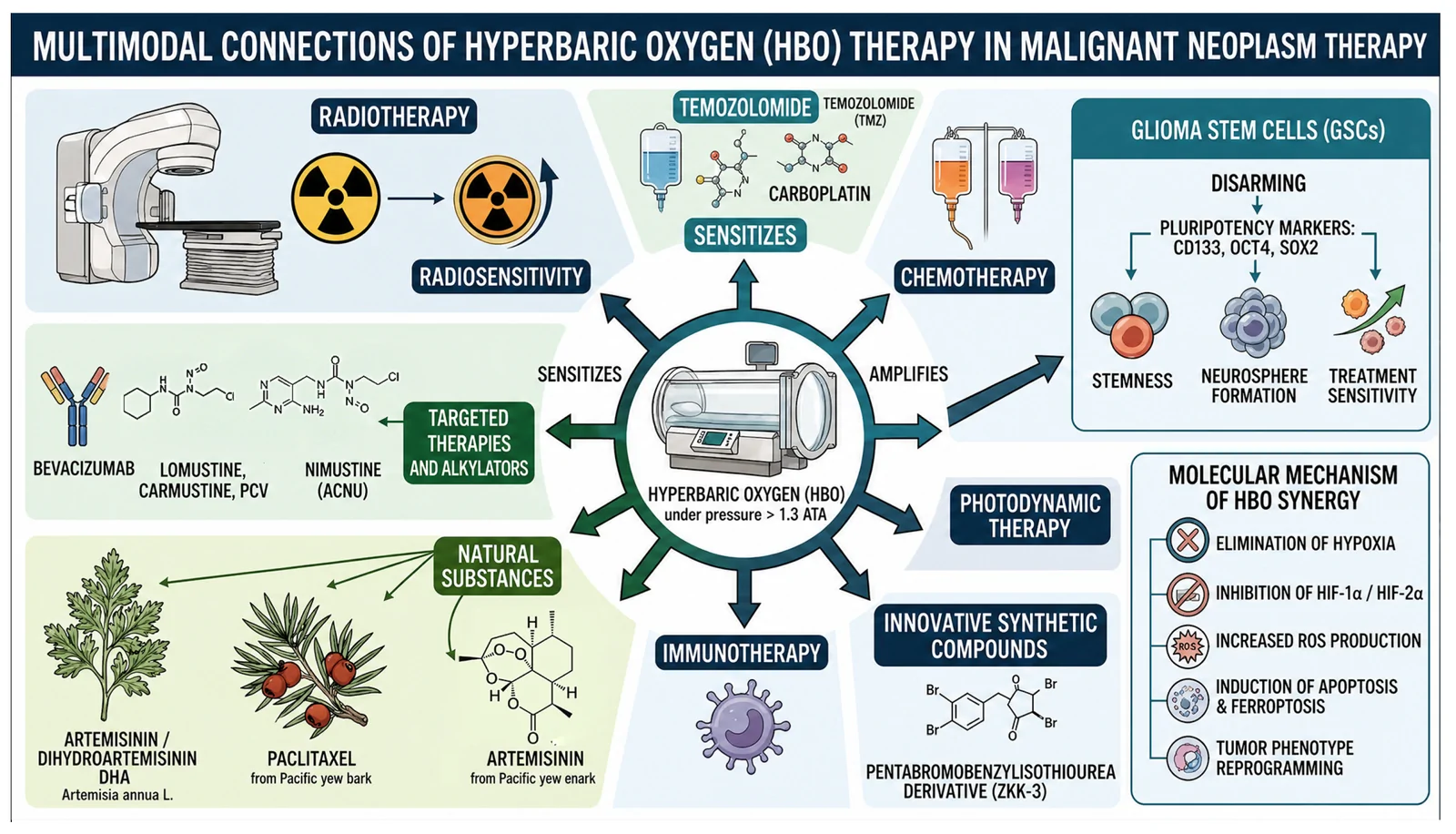
Multimodal effects of hyperbaric oxygen (HBO) therapy in malignant glioma treatment. HBO therapy serves as an adjuvant to enhance the therapeutic efficacy of both standard and innovative oncological protocols. HBO increases tumor cell sensitivity to radiotherapy (radiosensitization) and chemotherapy [65,66,67]. These include lomustine, carmustine, and nimustine, which are chloroethylnitrosoureas acting as alkylating agents, like temozolomide [68]. The molecular mechanisms underlying HBO sensitization include reducing intratumoral hypoxia, suppressing hypoxia-inducible factors (HIFs), intensifying oxidative stress (ROS production), inducing programmed cell death pathways (apoptosis and ferroptosis), and exerting targeted effects on cancer stem cells [69,70,71]. Furthermore, HBO facilitates pharmacological potentiation by enhancing the efficacy of natural substances (e.g., artemisinin, paclitaxel), targeted therapies (e.g., bevacizumab), innovative synthetic compounds (e.g., ZKK-3), and immunotherapies [64,72,73]. This figure was created using Affinity Designer, ChatGPT, and Gemini AI.

**Figure 2:**
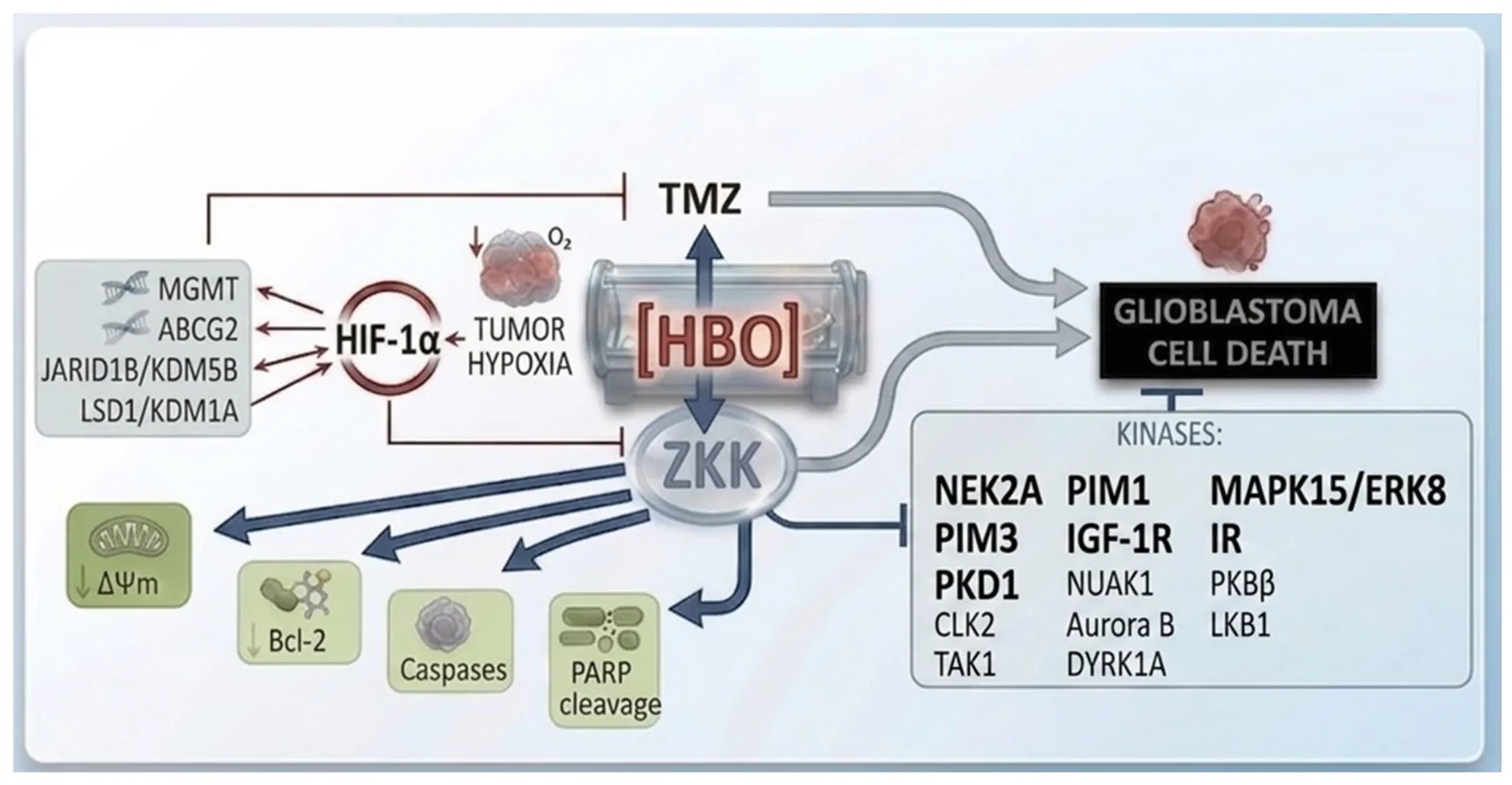
ZKK with TMZ and HBO as an investigational combination treatment for glioblastoma. The active form of TMZ methylates DNA, causing DNA damage and leading to tumor cell death. HBO supports TMZ by suppressing HIF-1α and its downstream targets involved in repairing TMZ-induced DNA damage [65,70,145]. ZKK3 inhibits numerous kinases that promote glioma cell survival, proliferation, stemness, and therapy resistance, thereby reducing residual activity to less than 50% and sometimes to less than 30% across a large panel of isolated kinases [127]. ZKK modulated glioma cell death, which was associated with caspase activation, proteolytic cleavage of poly(ADP-ribose) polymerase (PARP), and concurrent mitochondrial membrane depolarization [124]. Additionally, ZKK decreased the bcl-2 protein expression level [128]. Hyperbaric oxygen (HBO) is an effective method for relieving hypoxia associated with glioma [70]. *ABCG2* of the ABC transporter family, as well as *MGMT*, coding for a DNA repair enzyme, are HIF-1 target genes to which HIF-1 binds [27,28]. KDM5B (JARID1B), also elevated in glioblastoma, is upregulated by HIF-1α and contributes to mechanisms of TMZ resistance in glioblastoma cells from patients [143,144]. Additionally important is Lysine-Specific Demethylase 1 (KDM1A/LSD1), which has recently been implicated in cancer resistance by multiple mechanisms, amongst others by increasing hypoxia inducible factor-1a (HIF-1a)-mediated transcriptional activation due to its demethylation [137,138,139]. In particular, LSD1, highly expressed in GBM, plays a major role in GBM resistance to TMZ [140]. HIF-1alpha is also responsible for protecting glioblastoma at the nucleotide level by inhibiting MSH2 and MSH6, thereby avoiding futile mismatch repair cycles [29,30,31,146]. It is believed that the combined action of these therapeutic elements will yield greater effectiveness in targeting HGG tumors. The impact of ZKK on DNA damage mechanisms and DNA repair enzymes requires further study. This figure was created using Affinity Designer, ChatGPT, and Gemini AI.

**Figure 3:**
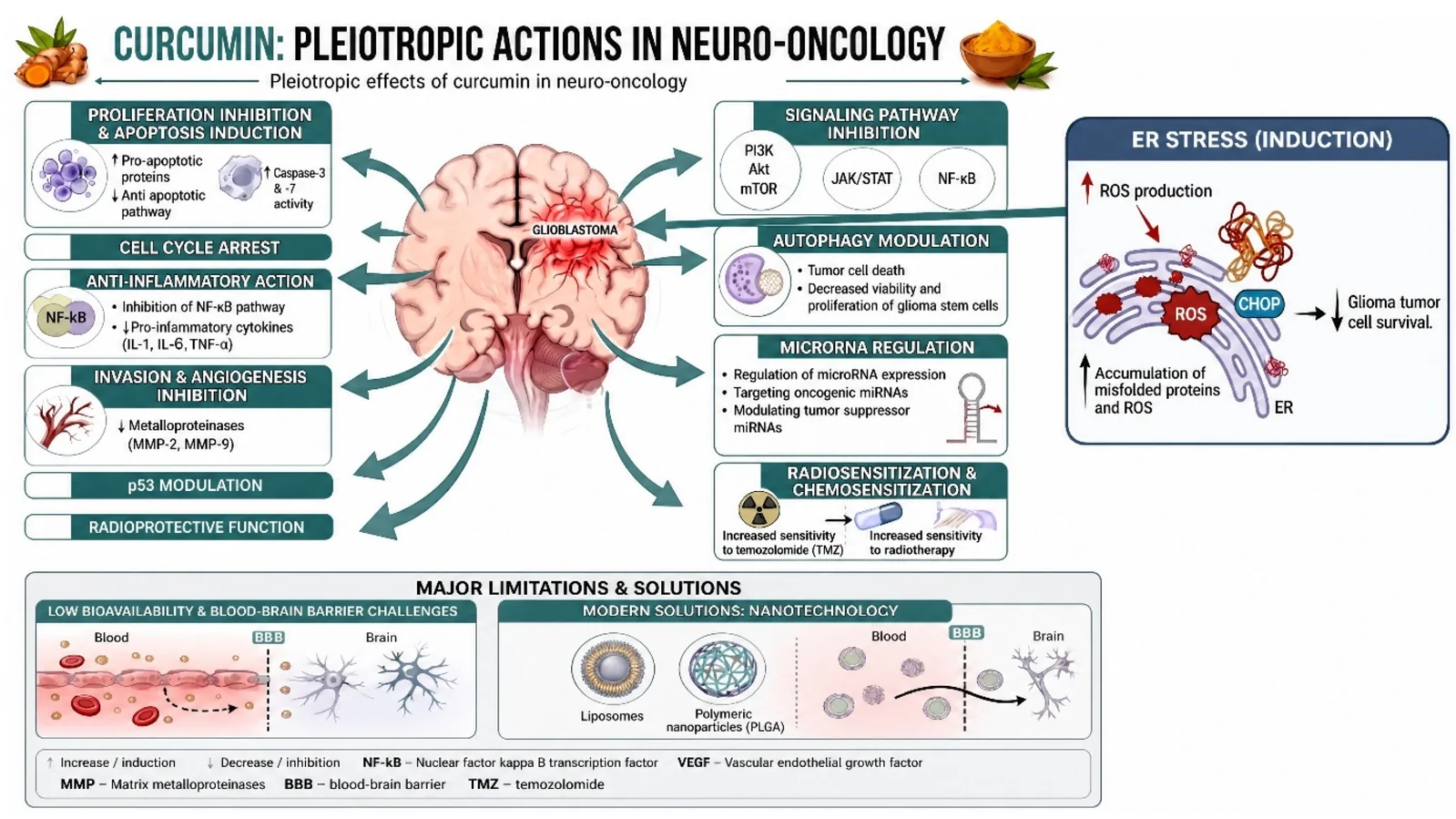
The pleiotropic effects of curcumin in neuro-oncology. Curcumin exhibits pleiotropic actions in neuro-oncology by targeting multiple signaling pathways—including NF-κB, JAK/STAT (Janus kinase/signal transducers and activators of transcription), and mTOR (mechanistic target of rapamycin)—to effectively inhibit glioblastoma growth [163,164,165]. It triggers cancer cell death by inducing Endoplasmic Reticulum (ER) stress, leading to lethal accumulation of reactive oxygen species (ROS) and misfolded proteins [165]. Beyond direct inhibition, the compound acts as a potent sensitizer, enhancing the vulnerability of brain tumor cells to standard radiotherapy and chemotherapy, including Temozolomide (TMZ) [166,167]. Curcumin further hinders tumor progression by suppressing angiogenesis and inhibiting enzymes involved in cancer cell invasion [164,168]. Additionally, curcumin regulates key cellular processes by modulating the p53 protein to restore apoptotic pathways, providing radioprotective effects in healthy tissues, and regulating microRNA expression and autophagy to control cell turnover [169,170,171]. Additionally, curcumin induces cell cycle arrest [167].To overcome the inherent challenges of low bioavailability and the blood–brain barrier (BBB), modern medicine employs advanced nanotechnology, such as liposomal delivery systems, to ensure the effective transport of curcumin directly to the brain [164]. This figure was created using Affinity Designer, ChatGPT, and Gemini AI.

**Figure 4:**
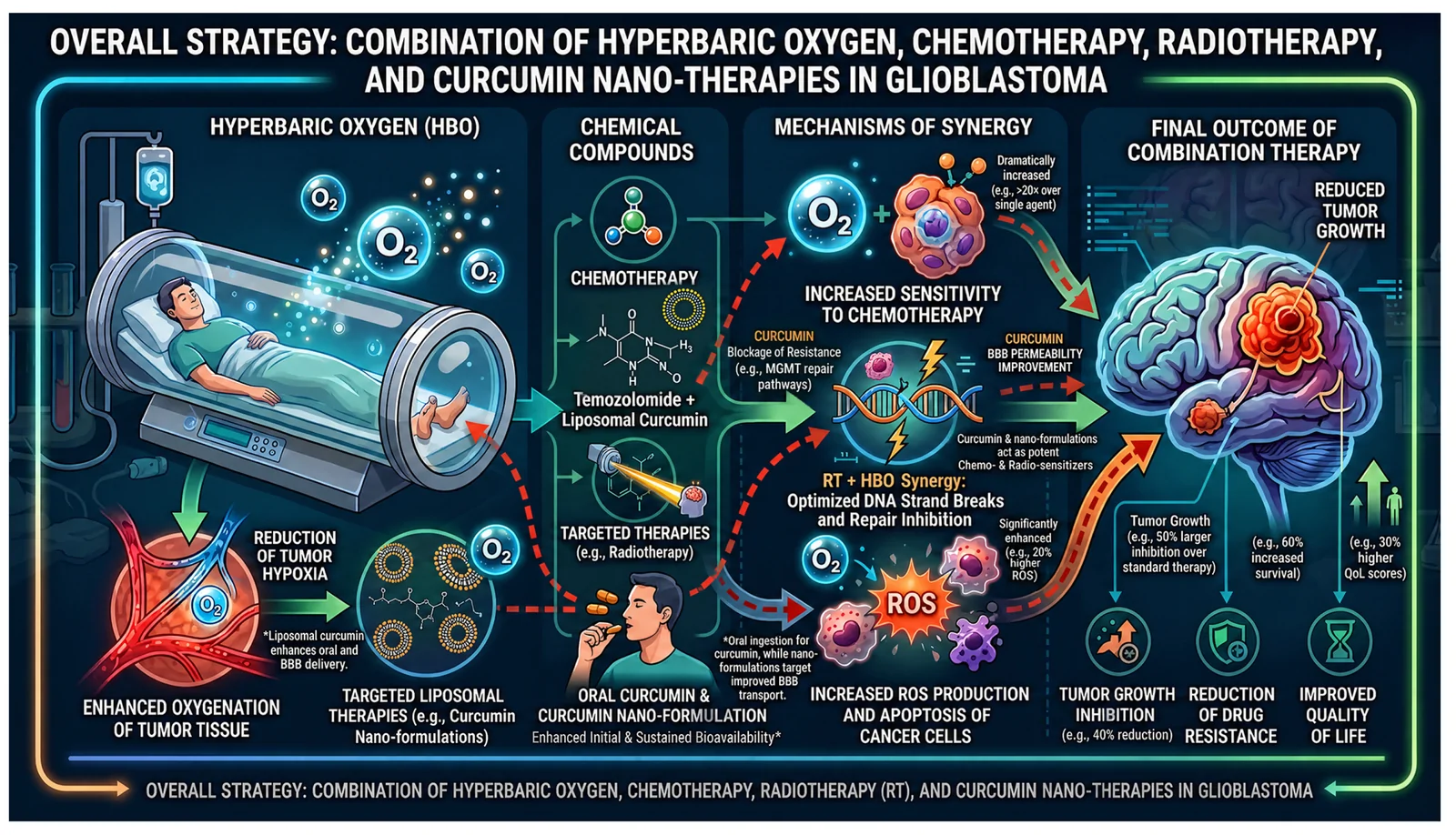
Therapeutic potential of curcumin combined with HBO and standard treatment for glioblastoma. The proposed approach integrates four core components: hyperbaric oxygen therapy (HBO), chemotherapy with temozolomide, radiotherapy (RT), and nanotechnology-enhanced curcumin formulations. Its mechanisms primarily focus on elevating oxygen levels within the tumor microenvironment to combat hypoxia, which impairs radiotherapy and chemosensitivity. Utilizing nanotechnology, such as liposomal curcumin, facilitates crossing of the blood–brain barrier (BBB) and boosts curcumin’s bioavailability, thereby enhancing its chemosensitizing role [179,188,189]. When combined, these methods may inhibit DNA repair processes, amplify ionizing radiation-induced DNA strand breaks, and elevate reactive oxygen species (ROS) levels, thereby promoting cancer cell death [165,179,190]. Overall, this strategy seeks to counteract glioma drug resistance and achieve statistically significant improvements in treatment outcomes. * Refers to a biphasic release profile: a rapid initial onset of therapeutic drug concentration followed by controlled, long-term sustained release. This figure was created using Affinity Designer, ChatGPT, and Gemini AI.

**Table 1:** Hyperbaric oxygen (HBO) therapy protocols used in combination with standard and investigational treatments for glioblastoma and other selected types of cancer.

Primary Treatment	HBO Treatment Regimen	Type of Study	Study Outcomes	Reference
Radiotherapy+ nimustine or ranimustine	60 min of 100% oxygen inhalation using an oxygen mask at 2.5 ATA daily	Clinical, malignant gliomas	tumor reduction of >50%, prolonged survival from 12 to 24 months	[86]
Carboplatin	0.2 MPa for 60 min, repeated at 24 h and every 6–8 weeks	Clinical, malignant gliomas or brainstem gliomas	HBO therapy prolongs the biological residence time of carboplatin	[67]
Radiotherapy + procarbazine, nimustine, and vincristine	30 to 60 min of 100% oxygen inhalation using an oxygen mask at 2.8 ATA followed by RTx within 15 min after exiting the chamber	Clinical, HGG	Combination therapy is safe, with virtually no late toxicities for high-grade gliomas.	[87]
Radiation therapy, TMZ	60–90 min in a monoplace HBO chamber pressurized with 100% oxygen to 2.0 ATA, ending < 15 min before RTx	Clinical, glioblastoma	The combined therapy was feasible without severe toxicities and proved to be a promising modality with longer OS times.	[88]
Chemotherapy, Curcumin	once-weekly course of hyperbaric oxygen therapy (90 min at 2 ATA)	Clinical, myeloma	Disease stabilization and good QoL	[89]
Stereotactic radiosurgery (SRS)	HBO at 100% oxygen and 2.4 ATA for 30 min; end of HBO session fell within 15 min before SRS delivery	Clinical, brain metastases	At 1 year, the estimated survival rates trended in favor of SRS + HBO	[90]
Radiation therapy	0.2–0.25 MPa for 90 min before RTx	Clinical, Single brain metastasis	Increased rate of remission while decreased rate of progression, improved QoL	[91]
Standard HGG therapy	1.35 ATA	Clinical, recruiting	t.b.d.	[92]
Artemisinin	100% O_2_ at room temperature and 3.5 atm for 90 min	Experimental; human leukemia cells	With HBO, an additional 22% decrease in the growth of human leukemia cells compared to artemisinin alone.	[93]
Temozolomide	An 80 min HBO (0.2 MPa) treatment was given three times at 12 h intervals	Experimental, U251 malignant glioma cells	Synergistically inhibited growth and induced death of cultured glioma U251 cells	[65]
Nimustine	2.5 atm pressure in a 100% O_2_ environment, for 90 min	Experimental, mice bearing a human glioma model (SU3 cells)	By the end of the trial, the tumor weight was lowest in the HBO + ACNU group	[94]
High-dose ascorbic acid (AA)	Cells were treated with one session of HBO (100% O_2_, 60 min, 2.5 ATA)	Experimental, VM-M3 cells	HBO significantly enhanced the cytotoxic effect of 0.3 mM AA	[95]
Doxorubicin-loaded liposomes(Doxil)	2.5 atm for 90 min	Experimental, hepatocellular carcinoma mouse model	Combination treatment synergistically inhibits H22 tumor growth in tumor-bearing mice over Doxil alone	[82]
TMZ-loaded porous silicon nanoparticles	2.5 atm for 2 h/day on days 1, 3, and 5 after injection of TMZ NPs (Nanoparticles)	Experimental, Rat glioma C6 cells injected in mice	Inhibition of tumor growth by TMZ NPs combined with HBO was observed compared with other groups on day 22	[77]
ZKK-3	2 ATA was applied for 1 h post-ZKK-3 treatment	Experimental, T98G glioblastoma cell line	HBO significantly reduces malignant glioblastoma cell proliferation while increasing sensitivity to the isothiourea derivative ZKK-3	[72]
Photothermal therapy (PTT) with nanoparticles loaded with TMZ	2.5 atm for 90 min	Experimental, NCH-421K and C6 glioma cells in vitro and in vivo	Combination treatment exerts significant antitumor effects (increased tumor inhibition rate) in mice bearing C6 tumors compared with PTT or TMZ NP alone	[96]
Radiotherapy and temozolomide	1.5 atm for 1.5 h every day up to 72 h for cells in vitro or on days 1–21 after tumor cell injection.	Experimental, GBM8401 and T98G GBM cell lines and NU/NU nude mice	HBO reduced the tumors’ ability to form cancer stem cells. HBO therapy increased GBM sensitivity to TMZ and radiotherapy both in vitro and in vivo.	[71]
TMZ	2.5 atm for 2 h	Experimental, primary glioblastoma cells and U87 and A172 cells, BALB/c-nude mice bearing tumor	HBO therapy combined with TMZ treatment significantly reduced tumor size and extended survival over TMZ alone. HBO without TMZ promoted GBM cell growth.	[70,75]

## Data Availability

No new data were created or analyzed in this study. Data sharing is not applicable to this article.
